# The Endurance and Contestations of Colonial Constructions of Race Among Malaysians and Singaporeans

**DOI:** 10.3389/fpsyg.2019.00792

**Published:** 2019-04-16

**Authors:** Geetha Reddy, Ilka H. Gleibs

**Affiliations:** ^1^Department of Sociology, University of Groningen, Groningen, Netherlands; ^2^Department of Psychological and Behavioural Science, London School of Economics and Political Science, London, United Kingdom

**Keywords:** identity, multiculturalism, race, intergroup relations, postcolonial, politics

## Abstract

Psychological literature on race has discussed in depth how racial identities are dialogically constructed and context dependent. However, racial identity construction is often not compared across different socio-political contexts. By researching racial identity construction in three different multicultural countries, Malaysia, Singapore, and the United Kingdom, we examined how three racial identities, Chinese, Malay, and Indian, are constructed among Malaysians and Singaporeans in this qualitative study comprised of 10 focus group discussions (*N* = 39). We applied Dialogical Analysis to the data. This paper shows that both racial ingroups and outgroups constructed all three racial identities, with ingroups constructing their identities more heterogeneously compared to outgroups. Participants also engaged with colonial constructions of the three racial identities. The geographical locations, and therefore their perceptual contexts, of the participants differed. Yet, colonial constructions of race endured in contemporary identity construction and were contested in the group settings. We conclude that the socio-political context as understood by the context of colonialism and post-coloniality influenced their racial identity constructions. Participants, regardless of differences in geographical location, used similar colonial constructions of Malay, Chinese, and Indian identities to position themselves as well as Others in their group interactions. These findings show that there is value in conceptualising the context beyond that which individuals are immediately presented with, and that psychologists should consider the inclusion of cultural legacies of colonialism in their conceptualisation of the present context.

## Introduction

“I would feel that I am definitely most, very proud to be Indian, especially when I’m overseas. Why I don’t know lah. Maybe because a lot of colonialism has rubbed into me, what I have read, so I’m very, a little, against it. (…) But when *you* subject me to some kind of, um, you know, social status where *you* look down upon me or something like that, if I get a feel of it, the Indian in *me* will come to the fore.”

–Shan, Singaporean, self-identified Indian

Racial identity is constructed, and reconstructed by individuals in the presence of Others – implied, imagined and real. The process of constructing a racial identity has been described, as “you think therefore I am” (Markus, 2010; p. 361), a twist on Descartes’ famous statement. It is therefore a dialogical process – one that is social and includes multiple voices. Indeed, the dialogical Self^1^ is oppositional to the Cartesian Self in that the former is embodied, tied to a specific place and time, and not only a self-contained individual (Hermans, 2001). Following this dialogical perspective, this article assumes that identity construction occurs when people engage in a collaborative meaning making of themselves within their social worlds. Thus, the Self is fundamentally relational – Others form part of the Self (Bakhtin, 1981). As seen in the quote above, the influence of the Other makes the Indian identity salient for Shan, and her racial identity becomes especially salient when she is outside of the country where she lives. Consequently, a social and cultural psychological perspective on racial identity construction should focus on how an individual’s construction of race draws from and feeds back to the social groups within which these constructions are made. It should also focus on how multi-cultural contexts influence these constructions and how harmonious or conflicting constructions among different individuals are managed or reconstructed. As such, this paper looks at the how racial identities are constructed, re-constructed and thus change, in group settings among Singaporeans and Malaysians living in Singapore, Kuala Lumpur, and London.

## What Is Identity?

Firstly, we argue that identity is not merely a product of memberships of different social groups (Tajfel and Turner, 1979) but rather a dynamic and contextualised process of connecting with a group, enacting that group’s representations and being viewed as a member of that group. We follow Duveen, (2001 p. 182) conception of identity which highlights that “identity is as much concerned with the process of being identified as with making identifications” and that “identities provide ways of organising meanings so as to sustain a sense of stability.” Indeed, the presence of others is important for individuals to develop the ability to recognise themselves, to build relationships with others, and to become self-conscious and agentic (Howarth, 2002). However, it is through social processes that the ‘contents’ (e.g., norms and values) of any identity are constructed. Group identities are made and remade in, and through, argument and social practise (Hopkins and Reicher, 2011). Keeping in mind the perception of others, multiple motives such as self-esteem, efficacy, continuity and meaning, influence identity construction (Vignoles et al., 2006). Some complex identities, like religious identities, are strategically constructed based on essentialist, politicised discourses to meet different needs within a community, such as promoting political action (Kahani-Hopkins and Hopkins, 2002; Hopkins and Kahani-Hopkins, 2004). The Self is also responding to the voices of Others. Individuals are motivated to understand what other people think and say, and often repeat or paraphrase the words of others (Marková, 2003; Gillespie and Cornish, 2010). Therefore, this Self-Other relationship is integral in understanding the process of identification, the content of the identity, as well as the motivations of the identity constructed.

Secondly, most identity researchers highlight the fluid nature of identities by stating that these identities are “constructed on the spot to reflect contemporary properties of self and others” (Haslam et al., 1992, p. 5). This is to say that identity construction should be firmly rooted in the immediate perceptual context – that is, the context that is present at the point of direct observation. Thus, the psychology of identity construction should be studied across different contexts to understand the differences among individuals who belong to the same categories and construct the same identities. This assumption drives this study.

While identity is best understood in the context that it is constructed and managed in, as many identity theorists argue (Howarth, 2002; Stevenson and Muldoon, 2010), context itself remains an allusive concept in many studies. The definition of context is often unclear and open for interpretation by the reader. Cornish (2004) concretised context in the psychological study of sex workers and health outcomes by focusing on moments where social phenomena are activated. She reduced context to specific time points where psychological processes take place. Research elsewhere has made the case for grounding psychological processes within a broader perspective of the socio-political context that is constructed and maintained by political elites (Verkuyten, 2013), and influenced by institutions (Andreouli and Howarth, 2013). Therefore, we situate our study of identity construction explicitly in group settings (specific moments) across different socio-political contexts (as demarcated by different geographical contexts). This approach can clearly capture the influence of socio-political contexts on racial identity construction, and the dialogicality of construction of identities among individuals.

To elucidate the process, content, and motivations of identity construction in its socio-political context we use the Social Representations Approach (SRA; Elcheroth et al., 2011). SRA combines both Social Identity Theory (SIT; Tajfel and Turner, 1979) and Social Representations Theory (SRT; Moscovici, 1984, 1988) and has been used together to understand socio-psychological processes that are embedded within a political dimension. Broadly, SIT helps understand the motivations of identity construction and negotiation, while SRT is focused on the process and content of the identity that is constructed. Four key assumptions of SRA are crucial for this paper. Firstly, social representations are shared knowledge that define how people act within their social worlds. Secondly, social representations are meta-knowledge -implying that the individual is reflexive and takes into account what one thinks that Others know, think and value (Elcheroth et al., 2011). Next, social representations are enacted communication that is shaped by factors that limit social practises, such as how others act toward us. Lastly, social representations are world-making assumptions that both constitute reality and, at times, change reality as well. In addition, we consider Staerklé et al. (2011) fifth component of SRA that seeks to show how shared knowledge is structured through “thinking in antinomies” (p. 762), which is the notion that thought is inherently dialogical. Thus, SRA invites the researcher to look at relations, rather than isolated individuals. What needs to be explored within the SRA paradigm, however, is how individuals use meta and shared knowledge to change the content of representations, and subsequently their racial identities.

### Racial and Ethnic Identities

Race, ethnicity and nationality are important social categories for many individuals. They form part of an individual’s self-concept that the individual adopts to make sense of their social worlds (Billig, 1993; Shih et al., 2007). Constructions of race are meaningful for minority group individuals living in multicultural societies across the world (Luke and Carrington, 2002; Verkuyten, 1997). Influential work by Clark and Clark (1947) firmly grounded the importance of the psychological study of the impact of race on everyday lives of individuals. Since then, psychologists have focused on the different ways that race impacts upon an individual’s sense of wellbeing (Townsend et al., 2009), attitudes on immigration (Deaux, 2006), sense of belonging (Howarth et al., 2013), views on multiculturalism (Verkuyten, 2001), experiences of colonisation (Fanon, 1967), intergroup contact (Ramiah et al., 2015) and, evident in all these studies, racism (Tizard and Phoenix, 2002). Often, the focus has been placed on how individuals and relevant Others identify themselves as members of their racial ingroup.

Whilst a very salient aspect of the lived experiences of racially marginalised and disempowered groups around the world, academic literature that is often published in many “mainstream” psychology journals engages little with institutional prescriptions of race on individuals and how these prescriptions influence on the psychology of these individuals (but see Sellers et al., 1997). This is not to say that such academic work does not exist. On the contrary, thorough research has been conducted, and continues to be conducted, on the effects that institutions have on racial identity. However, such discussions are often more prevalent in what are considered “niche” journals, such as the Journal of Black Psychology (Utsey et al., 2015) and Cultural Diversity and Ethnic Minority Psychology (David and Nadal, 2013), in the realm of applied psychology, such as Journal of Community and Applied Social Psychology (Durrheim and Dixon, 2010) or in medical sociology, such as Journal of Health and Social Behaviour (Sellers et al., 2003). This article contributes to the endeavour of increasing visibility of these lived realities of individuals within academia. We also hope that the open-access policy of Frontiers in Psychology would result in a greater engagement with our work and that of others we cite here.

We have decided to use the term race throughout the article reflecting the manner with which it is used by our participants and is constructed in governmental discourse. We take Brah (1996) position that race and racism are dynamic social processes that are different in different social contexts. In Malaysia and Singapore, the terms race and ethnicity are used interchangeably, with race being more commonly used in general public debates. In these two countries, race is understood to be patrilineal and inherent in one’s biological makeup. Furthermore, scholars have acknowledged that the sole focus on ethnicity has left the persistent nature of racism unaddressed (Harrison, 1995). In understanding intergroup relations in a context where race is a meaningful category, we believe that it is imperative to use terms that reflect the current discourse. As such, race is used in this paper without the use of double quotes so as to reflect its use in the context of Singapore and Malaysia, specifically addressing or reflecting government or participants’ discourse. However, from our perspective it is understood as being socially constructed, situational and fluid, and not a biological fact.

It is widely accepted within the social sciences that race is not related to one’s biology. However, the everyday understanding of race is that it is entrenched in inherent differences. It is this disconnect, between the academic understanding of race and the everyday understanding of race, that makes the contexts of Malaysia and Singapore important and interesting research contexts to study racial identity construction. Political scientists and sociologists have been interested in the multicultural frameworks used in these two countries because of their impact on political ideologies, and development of civil societies. Multicultural societies such as Malaysia and Singapore present a unique opportunity for psychologists to understand how an individual’s everyday engagements with race-based policies influence how they make sense of themselves and their social worlds because they provide a sharp contrast to contexts that do not explicitly utilise race-based social policies.

While there is extensive research and publication on race in the Western world, there is much less academic engagement with constructions of race beyond the West. Even as we avoid the dichotomy of East versus West, collectivistic versus individualistic cultures, cultural differences do lead to different construals of the Self and Other (Markus and Kitayama, 1991). Indeed, there is a rich tradition of psychological studies from Asia, Africa and Latin America that inform our understanding of the human condition and that cannot be ignored (Sinha, 1981; Paranjpe et al., 1988). There have been many, more recent, contributions to the study of racial identities from South Africa (Bowker and Star, 2000), Malaysia (Gabriel, 2015), and Brazil (Bianchi et al., 2002), for example, which have used and extended Western research and theories on racial identities. To this end, there are many insights to be gleaned from directing our focus to racial identity constructions in a comparative study among Malaysians and Singaporeans. Thus, in line with understanding racial identity construction across contexts, three different socio-political contexts, Malaysia, Singapore, and the United Kingdom, were selected.

## Malaysia, Singapore, and the United Kingdom (Uk): an Overview

Many scholars have documented the indelible marks left by colonisation that shapes many aspects of the daily lives of individuals across the world (Fanon, 1967; Said, 1979; Adams and Salter, 2007; Hilton, 2011). Colonisation has especially influenced the psychology of individuals with regard to race and culture (Okazaki et al., 2008). The connections between knowledge, power and practise have impacted the construction of colonised subjects (Mama, 1995). Malaysia and Singapore which were ruled as one entity (Malaya) by the British until 1959, form an important part of our study as they show how the nations’ evolution, influenced by colonial rule, sets the socio-political context for the construction of identities. Specifically, colonial adminstrators sought to define the diverse population by racial categories that roughly related to the different countries that individuals migrated from, and were based on anthropological constructions of indigenous populations (Reddy and Selvanathan, in press). The postcolonial Malayan government desired a multicultural nation united by a single language – Malay – and the idea of a Malay Malaya was central to this ideology. However, this policy faced resistance by non-Malays who perceived it as a threat to their own cultural identities and practises. Race riots between Chinese and Malays in Singapore in 1964 (and later in 1969) were believed to be caused by differences in political ideologies in the two countries (Noor and Leong, 2013). Postcolonial Malaya thus separated into Singapore and Malaysia amidst racial tension on economic, social and political fronts.

Two different models of multiculturalism developed in the two countries (Noor and Leong, 2013), forming two distinct socio-political contexts; yet both postcolonial goverments maintained colonial racial categories. Malaysia’s *‘Bumiputera’ (sons of the soil)* policy means that non-Malay Malaysians accept Malay supremacy in exchange for citizenship. In Singapore’s policy of multiracialism that is guided by pragmatic realism and economic goals, the ‘social formula’ of the CMIO model is built upon the acceptance of the four main races in Singapore – Chinese, Malay, Indian, and ‘Other’ (CMIO) – as separate but equal in formulating most of its social policies. This policy positions Singapore as a meritocracy. However, the reality is that a focus on individual race-based cultural development and differential opportunities has led to unequal power dynamics amongst the population, resulting in racial inequalities (Chua, 2005; Mutalib, 2012). In this study we present a cross-country comparison of three racial identities (Chinese, Malay, Indian), present in both countries, that were created through a joined history of migration, historical racial boundary-making and contemporary institutional social policies.

When understanding racial identity construction in the contexts of post-colonial Malaysia and Singapore, it is imperative to look at the process of racial categorisation. The categorisation process has both administrative and informational functions in understanding the perceived world (Bowker and Star, 2000). Categories create the idea that the world is structured into predictable attributes, rather than arbitrary ones, thus maximising information with least effort on the individual’s part (Rosch, 1978). However, informal and formal categorisations of the same object may have different contents and meanings. For example, the formal categorisation of race in Singapore and Malaysia involves a classification of an individual based on her/his father’s racial categorisation via patrilineal determination. This formal categorisation takes place at birth, being inscribed in the birth certificate and national identity card of all individuals born in the two countries. A visible representation of both racial and national identity is presented together for the individual. Futhermore, Malaysians and Singaporeans are encouraged to identify with hyphenated identities that encompass both their racial and national identities such as Malay–Singaporean and Chinese–Malaysian (Hashim and Tan, 2009). Racial and national identities are thus often constructed together in the formal categorisation of the individual.

That this formal categorisation process has its roots in British colonial management of diverse populations is relevant because British colonial strategies of ‘define and rule’ were created to determine people’s function in the colonial economy (Mamdani, 2012). Indians were mainly recruited to work as ‘coolies’ in plantations, Chinese peasants were segregated in the tin mines and the local Malay peasantry were largely left bound to their rural-based activities (Hua, 1983). This separation meant that the heterogeneity within the diverse populations was collapsed into simplified racial categories for ease of administration. Not only did colonial adminstrators stratify the population administratively, they also attributed specific characteristics to members of each racial category. These colonial constructions of race were even detailed in handbooks for new European residents so that they would know how to engage with local populations (Harrison, 1929). In the text below found in one such handbook, we see colonial constructions of members of the Chinese racial category.

*“The Chinese community...ready to learn, obliging, full of zeal, but rich in nothing but a sound mind in a sound body, shrewd and strong. Sometimes, of course, he comes masquerading as this, but is in reality a pirate or a gang robber to his trade.”* (Harrison, 1929, p. 54).

Postcolonial governments of the two countries carried forward formal categorisations of race from the British. From a political perspective, little has changed since independence from colonial rule with regards to the importance of the racial categories as well as the content of these categories in Singapore (see Reddy, 2016, for an elaboration). Race has retained its role as the prime apparatus of administration and control, with race-based political parties in Malaysia deriving their origins and ideologies from post-colonial context (Gabriel, 2015). Yet perhaps what is less studied, within the contexts of Malaysia and Singapore, is whether these colonial constructions of race persist in contemporary construction of racial identities given that the colonial racial categories remain in formal racial categorisation today.

On the other hand, appearance, language, and participation in that racial group’s life constitute an informal, vernacular categorisation of race in the two countries. These vernacular categorisations tell us about what people do with formal categorisations. Interactions occur between the informal and formal categorisations, showing us that we should aim to find out how people place themselves and Others into categories situationally (Edwards, 1998). Singapore and Malaysia highlight how through two classification systems, the concept of race is kept alive and used to hold institutions and people together (Desrosières, 1990). The formal and informal converge in the Singaporean and Malaysian individuals’ everyday engagement with social policies and in their interactions with one another.

While colonial rule reified racial categories used both formally and informally, social and cultural psychology has been at pains to understand disruptions and changes to such categories. As Gillespie et al. (2012) argue, social categories are perspectival (rooted in a social position), historical (changing categories, and changing human groups), disrupted by the movement of people (people move in and out of social categories), and re-constitutive of the phenomena they seek to describe (reproducing categories in theory leads to reifying them in practise). By applying this perspective to Malaysia and Singapore, we need to keep in mind what the categories of race mean within the specific historical, political and geographical context. At the same time, it is necessary to understand if and when individuals can move in and out of these categories that have been placed upon them.

Although in Singapore and Malaysia today, racial identity is used to allocate resources such as education, housing and employment, and is assigned by the state, race is constructed very differently by the United Kingdom state. Here, individuals have the option to choose an ethnic (not racial) label for themselves such as White and Black Caribbean at the institutional level, and assigning resources based on race would be considered illegal racism. We thus chose London as a research site because thousands of Singaporeans and Malaysians take up temporary or permanent residence in London (Office of National Statistics, 2013). London presents an interesting research context to study how Malaysians and Singaporeans construct race as they would not need to use racial categories imposed by the Singaporean and Malaysian government to access resources in the United Kingdom, and have the option of giving themselves a racial identity with which they self-identified. Therefore, the assumption is that Malaysians and Singaporeans living under different socio-political contexts (such as the United Kingdom) would construct Malay, Chinese, and Indian racial identities differently because the identity construction process is mediated by the immediate perceptual context.

### Present Study

We focused our research on the construction of Malay, Chinese and Indian racial identities in three different socio-political contexts: Malaysia and Singapore where race is constructed by the state and plays a salient role in the way individuals interact with social policies; and the United Kingdom where race is self-constructed at the institutional and formal categorisation level. We studied if there was a difference in the construction of the same racial identities by Malaysians and Singaporeans when they had experiences of living outside of the two countries of origin, especially in a country that does not utilise similar race based social policies. This study forms part of a larger study exploring the connection between context and racial identity construction. Thus, the research question for this study was: *How do different socio-political contexts, namely Malaysia, Singapore, and the United Kingdom, influence racial identity construction among Malaysians and Singaporeans in group settings?*

## Materials and Methodsology

To understand issues of race and race relations, it is necessary to use methodologies that will ground the research in the everyday experience and talk about these experiences (Durrheim and Dixon, 2005). Thus, focus group discussions and Dialogical Analysis (DA) were chosen to enable a clear understanding of the multiple meanings that categories hold for individuals. Michael Billig criticised how identity theorists analyse identities without any distinction between laboratory settings and categories that have meaning outside of the laboratory. This critique concluded that meanings associated with social groups were more important for the social identities of people than how an individual self-categorised (Billig, 1995). Therefore, we decided to host focus group discussions among the participants so as to reflect the society at large, given Farr et al. (1996) view that focus groups are “thinking societies in miniature” (Lauri, 2009, p. 650). We applied DA with a focus on metaperspectives within the intersubjectivity paradigm (Gillespie and Cornish, 2010) and multivoicedness (Aveling et al., 2014) to the data. Social and cultural psychologists have used intersubjectivity to study the context within which interlocutors make meaning (Jovchelovitch, 2007). The dialogical approach allowed us to unpack the multiplicity in the constructions of Self (identity) and Others (and how the Other is embedded in the Self), where we focused on how Others influence the self-construction of racial identities. We went beyond the purely individualistic approach to identity construction and explored how participants co-constructed self-constructions of racial identities with Other, focusing particularly on the process, motivations and content of racial identity construction in each context.

We developed a semi-structured interview schedule that was used in all three locations (Singapore, Malaysia, and London). A review of the literature, discussed above, assisted in the development of the interview schedule as it allowed the interviewer (first author) to identify key topics that would be relevant to Malaysians and Singaporeans with regard to their racial identities. However, no questions pertaining to colonial history were asked in the main questions and prompts. Additionally, no references were made to the colonial history by the interviewer. The interview schedule is attached as Appendix A. It consists of nine open-ended, exploratory main questions, explanatory probes and a Vignette List. Some examples of these main questions are, “*What are the ways you explored your Malay/Indian/Chinese Identity*?” and “*How similar is being Malay/Indian/Chinese Identity in Singapore and London*?”. The explanatory probes were used as and when necessary. As this data was collected as part of a larger study, the vignettes functioned as prompts for a specific part of that study centred on multiracial identities, and are not the focus of this paper. Colonial constructions of Asian identities in the two countries did not address “mixedness” between and among the diverse Asian populations, only acknowledging intermarriage between European and Asian individuals (Rocha, 2014). In light of this limitation, multiracial identity construction was left out of this paper but addressed elsewhere (Reddy, 2018).

The research process was double hermeneutic in nature (Giddens, 1987) and researchers were reflexive in their approach, methodology and position when embarking on the study and analysing the results (Shope, 2006). The first author conducted all of the focus groups in the three different countries. Two aspects of her identity were particularly pertinent. The first was her positioning as a Singaporean, which became clear to both Malaysian and Singaporean participants because of her Singaporean accent. While participants spoke to her in various local languages it was the manner with which she spoke English that differentiated her from her Malaysian counterparts. Thus, participants positioned her as either an outsider or an insider based on participants’ own national identification. It must also be noted that participants often drew on the first author’s identity as a Singaporean when broaching topics that required an understanding of the Malaysian and Singaporean context. The second aspect of the first author’s identity that was salient in the research context was their multiracial identity, which participants discussed at the end of the focus group discussions. The first author was perceived to be either Indian or multi-racial by the participants. The second author identifies as white and coming from a Western European country. She was not present during the focus groups, and thus ‘invisible’ to the particpants and in the discussions.

### Participants

We conducted ten focus group discussions, with a total of thirty-nine participants, in three different locations – Kuala Lumpur (capital of Malaysia), Singapore and London (capital of United Kingdom). As discussions surrounding the topic of race were considered sensitive in Kuala Lumpur and Singapore, the groups were smaller than the ideal number for focus group discussions. Two focus groups that were intended to be carried out in Malaysia did not materialise because of unexpected attrition due to the timing of the focus groups. Focus groups in Malaysia were conducted 2 weeks after the introduction of the new Sedition Act in 2015 (Agence France-Presse, 2015) and we postulate that this law may have influenced participants’ willingness to participate. Confidentiality was emphasised and participants details were anonymised accordingly. To protect participant confidentiality, all interview participants were given pseudonyms during the transcription process.

Participants were recruited online via the social media platforms Facebook and Twitter. Participant recruitment advertisements outlined that the first author is interested in how Malaysians and Singaporeans understand ethnic identities and how they feel that their environment helps them in their understanding. Participants had a range of educational backgrounds. Only one out of the 10 focus groups was conducted with only university students (Malaysians in London, *n* = 5). There were no students in the focus groups conducted in Malaysia. All other focus groups had a mix of students from different educational institutions in Singapore, Malaysia, and the United Kingdom, and working adults. Focus group details are provided in Table 1.

**Table 1:** Focus group details.

Participant details	Malaysian	Singaporean
Mean Age	26.1 years	32 years
Female (*n*)	8	15
Male (*n*)	8	8
Focus groups in Malaysia	2 groups (*n*_1_ = 3, *n*_2_ = 4)	0
Focus groups in Singapore	0	4 groups (*n*_1_ = 5, *n*_2_ = 4,*n*_3_ = 3, *n*_4_ = 3)
Focus groups in London	2 groups (*n*_1_ = 6, *n*_2_ = 3)	2 groups (*n*_1_ = 5, *n*_2_ = 3)

All but three focus group discussions had participants who identified as mono-racial (Malay, Chinese, and Indian) and multiracial (Chinese and Indian heritage, for example). In two groups, all participants identified as mono-racial. In one group, all participants identified as multi-racial at different points in the discussion. It is of significance that all participants were categorised with only one racial identity but some identified as multiracial. Participant racial categorisation was captured at the start of the focus group discussions. These details are provided in Table 2.

**Table 2:** Participant background details.

Pseudonym	Age	Racial categorisation	Educational qualifications
**Singaporeans in Singapore**			
Kelvin	25	Chinese	Degree
Nadia	28	Indian	Postgraduate
Janet	25	Chinese	Degree
Kumar	33	Indian	Postgraduate
Shan	53	Indian	Postgraduate
Zainal	27	Malay	Diploma
Mika	37	Indian	Postgraduate
Jesslyn	31	Chinese	Postgraduate
Heera	27	Indian	Degree
Ben	28	Chinese	Degree
Saiful	31	Javanese	A level
Eugene	31	Chinese	Postgraduate
Aarif	36	Indian	O level
Nurah	34	Indian	Diploma
Helen	33	Chinese	Postgraduate
**Malaysians in Malaysia**			
Sarojini	33	Indian	Postgraduate
Sachin	24	Indian	A-level
Nirmal	35	Indian	Degree
Arvin	28	*Kadazan*	Degree
Sanjana	23	Indian	Degree
Zaza	28	*Bajau*	Degree
Anita	36	Ceylonese	Postgraduate
**Singaporeans in London**			
Sofia	41	Malay	Diploma
Zara	32	Malay	Postgraduate
Revathi	25	Indian	Postgraduate
Bala	25	Indian	Postgraduate
Ray	27	Chinese	Degree
Pam	59	Eurasian	O level
Jon	26	Chinese	Degree
Mel	22	Chinese	Postgraduate
**Malaysians in London**			
Ilan	34	Malay	Postgraduate
Aadil	23	Malay	Degree
Jing Wei	32	Chinese	Degree
Amit	21	*Lain-Lain*	A levels
Louisa	20	Chinese	A levels
Noel	21	Chinese	A levels
Christine	21	Chinese	A levels
Trina	20	Chinese	A levels
Selena	19	Chinese	A levels

The first author conducted the discussions in English. All participants were fluent in English and used phrases in three local languages- Mandarin, Malay, and Tamil – that the first author had at least conversational competency in. The discussions were digitally recorded and transcribed verbatim, and then translated to English. All discussions in London were conducted in university rooms, whilst all discussions conducted in Malaysia and Singapore took place in home settings. Participants in London were reimbursed with £5 for transport costs. Participants in Malaysia and Singapore were not reimbursed for transport costs but were served refreshments during the discussions.

#### Ethics

This study was carried out in accordance with the recommendations of the British Psychological Society (BPS) ethics guidelines and approved by the Chair of the Department of Psychological and Behavioural Sciences ethics committee, as well as Research Degrees subcommittee of the London School of Economics and Political Science, United Kingdom. All subjects gave written informed consent in accordance with the BPS ethics guidelines that refers to the Declaration of Helsinki. The protocol was approved by the Chair of the Department of Psychological and Behavioural Sciences ethics committee of the London School of Economics and Political Science, United Kingdom.

### Analysis

In scaffolding the analysis of the data, we asked the following questions of the data – *Who is (and from which position are they) constructing the racial identity? What entails this construction? How do they express this construction? How do Others interact with this construction?* and tabled these constructions. A sample of the data analysis process is given in Table 3.

**Table 3:** Sample of data analysis.

Quotation	Construction of Chinese Identity	Who is constructing? (and position)	How do they express this?	How do others in FGD interact with this?	Point of conflict/Dialogical knot?
A:The Chinese are very hardworking, very entrepreneurial, very crude, very sharp, very kiasu	Chinese as enterprising, Chinese as Kiasu	Anika, Indian position	Reflecting the voice of non- present Others in a discussion on stereotypes	No contestation from others	No point of conflict
Z: Like no mixture you know, pure Chinese K: So every part of them lah, every part of the animal	Chinese as eating every part of the animal	Zainal, Malay position; Kumar, Indian position	Drawing on stereotypes to establish common understanding of Chinese identity	Group laughs in acceptance, however, Mika expresses disappointment in negative construction	No point of conflict
E: the idea of Chinese identity probably doesn’t come to mind so strongly because Singapore is afterall a Chinese city. Yeah, so being majority, it’s sort of like it’s something you don’t contemplate on a daily basis as much as I would hear from my friends of other ethnicities…	Chinese as privileged	Eugene, Chinese position	Own perspective	No comment from group, move on to next topic	No point of conflict

This approach allowed us to first identify all ‘I’ positions and Other positions relevant to racial categories in the two countries in the text. For example, every time participants referred to a racial identity, we would highlight the text and mark if the participants was referring to their own racial identity or to that of another person. Second, we identified voices of the inner Others in discussions on racial identities. Here, we went through the highlighted texts to see if participants were claiming to express their own views or were drawing references to other people such as a parent’s point of view. We then examined the dialogue and relationships between the different voices, as suggested by Aveling et al. (2014). Multivoicedness is the simultaneous existence of different individuals’ voices in any individual. It is also the simultaneous existence of individual voices and the voices of groups (Bakhtin, 1981). Thus we focused our analysis and presented extracts here that showcase both dialogues between focus group members, but also within each individual. Essentially, we identified challenging sections of the focus group discussion where participants brought up a point of conflict or contention (a device called *dialogical knot* in DA) and resolved this conflict through their dialogical construction of the racial identities. These points of conflict can be connected to both positive and negative constructions becausetension need not arise only in the context negative representations of an identity^2^.

The transcripts were analysed both by hand and using NVivo. The first step was to read the transcripts several times and make notes by hand. In the second step, we used NVivo to identify relevant sections in the texts according to the questions outlined above. We then extracted these sections and analysed each section separately by hand. The dataset from each geographical location was analysed together first (Kuala Lumpur groups together, Singapore groups together, and London groups together) and then a secondary analysis was carried out where differences and similarities between the groups were compared. Our interpretation of the primary data was informed by other sources of information, such as newspaper articles about the socio-political contexts, as suggested by Aveling et al. (2014). The theoretical framework, knowledge about the socio-political contexts, and data continued to speak to each other in the analysis of the data, and were unpacked together, leading to meaning emerging as a joint creation (Sullivan, 2012). For example, in the period that we were identifying all the different racial constructions, we also engaged with literature on Malaysian and Singaporean history, and multiculturalism policies. This included academic articles that outlined colonial administrators reports. The analysis was structured around identifying how racial identities were constructed by both ingroup and outgroup members, as can be seen in Table 2. Analysis framework and codes were discussed in depth by a senior academic experienced in DA and the final coding framework was developed after extracts, relevant codes and ‘I’ positions were corroborated.

## Results and Discussion

Our findings will be discussed in two sections. First, we give an overview of the different constructions of Malay, Chinese, and Indian identities that emerged in the data, showcasing the breadth of the content of racial identity constructions among Malaysians and Singaporeans. Second, we connect these constructions to the processes and motivations of identity construction, demonstrating how these processes influence interactions and, by extension, intergroup relations between ingroup and outgroup members.

### The Content of Racial Identities

Based on our theoretical assumptions outlined above, we examined differences between Malaysians and Singaporean participants in the three different geographical locations. However, we found that constructions employed by participants in one location (for example, Singapore) were also shared by participants in other locations (for example, London). Constructions made by Malaysians were also brought up by Singaporeans. *Dialogical knots* were also present in the same constructions in all three geographical locations. In light of these three points, we broadened our analysis across ingroup racial identity constructions, and outgroup racial identity constructions. Malay, Chinese and Indian racial identities were discussed by both ingroup and outgroup members. In other words, Chinese identifying individuals constructed Chinese identity (ingroup) as well as Malay and Indian identities (outgroup). These individuals represented their own opinions, but also echoed those of their family members, and other racial group members, which demonstrates DA’s understanding of multiple positions (multiple voices).

Broadly, participants engaged in a wide range of constructions about their own and other racial identities across the three locations. These constructions ranged from identities being embedded in the languages being spoken (Chinese as Mandarin Language speakers, Indians as Tamil language speakers) to identities possessing qualities (Chinese as traditional, Malay as rich in culture, Indians as united) to physical appearance (Indians as black, Chinese as having small eyes). That participants’ constructions of outgroup racial identities was less heterogeneous compared to their constructions of ingroup racial identities is noteworthy. For example, Malay identifying individuals constructed Chinese identity only as “enterprising” and “privileged,” while Malay identity was constructed in a more diversified manner. This conception has been proposed previously by Tajfel (1981) when he showed that the outgroup is constructed to be more homogenous than it is, while the ingroup is constructed to be heterogeneous. This is also known as the ingroup heterogenity/outgroup homogenisation effect (Park et al., 1991).

Moving beyond an analysis of differences between geographical contexts meant that we could critically focus on the similarities between them. Specifically, when we examined the findings with a historical, colonial understanding, we could see that participants were engaging with similar stereotypical constructions. For the purposes of this paper, we draw on the following colonial constructions of the Malay, Indian, and Chinese identities. Colonial constructions of race were born from the imaginations of early European residents and administrators in British Malaya as mentioned before and summarised in this extract below:

“From a labour point of view, there are practically three races, the Malays (including Javanese), the Chinese, and the Tamils (who are generally known as Klings). By nature, the Malay is an idler, the Chinaman is a thief, and the Kling is a drunkard, yet each in his own class of work is both cheap and efficient, when properly supervised”

Wamford-Lock (1907, p. 31)

The colonialists’ denigration for the Chinese went even as far as, “Whenever money is to be acquired by the peaceful exercise of agriculture, by handicrafts, (…) there will be found the greedy Chinese” (Newbold, 1839, p. 10, in Hirschman, 1986). Whilst participants were not provided with these colonial constructions at any point in time during the discussions, these perceptions were clearly reflected in participants’ contemporary constructions of Malay, Indian and Chinese identities. Specifically, these are: “*Indians as alcoholics and labourers, Malays as lazy, Chinese as “kiasu”^3^*(see Table 4). Both ingroup and outgroup members engaged with these three constructions. For example, “Malays as lazy” was constructed by both Malay participants and Indian participants, while Chinese participants constructed Malay identity in a more nuanced and less negative manner by constructing Malays as not industrious and relaxed. We will return to this point later.

**Table 4:** Content of constructions.

Constructions by	Constructions of		
	Chinese Identity	Malay Identity	Indian Identity
Chinese identifying individuals	Chinese as a Mandarin Language speaker Chinese as traditional Chinese as enterprising Chinese as religiously diverse Chinese as educated in Chinese medium schools Chinese as kiasu Chinese as privileged Chinese as having small eyes Chinese have specific cultural values Chinese as a prescribed identity Chinese Malaysian different from PRC Chinese Traditional Chinese are different from Chinese Christians Chinese as a chopstick user Chinese as interested in Chinese culture Chinese as practical	Malays are relaxed and not industrious Malay as Muslim Malay as made to be complacent Malay as a Malay speaker Malays don’t eat pork and don’t drink Malays as not having certain military positions in Singapore Malays are treated unfairly	Indians as Tamil Language speaker Indians as well spoken Indians as doctors and lawyers Indians do not have to be Hindu
Malay identifying individuals	Chinese as enterprising Chinese as privileged Chinese as eating all animal parts Chinese as good with numbers Chinese as not skilled in arts	Malays who speak English have lost their Malayness Malay as lazy Malay as Muslim Malays as insular group Malay as a Malay speaker Malays are not homogenous Malay as *minah* Malay as rich in culture Malay and Islam is separate Malay through wearing Baju Melayu (traditional Malay clothes) Malay as becoming Arab Malay as having Hindu origin	Indians as Tamil Language speakers Indians as united Indians as North Indian and South Indian Indians as smelling of curry
Indian identifying individuals	Chinese as Mandarin language speakers Chinese as eating pork Chinese as enterprising Chinese as *Kiasu*	Malay as Muslim Malays don’t care about money Malays are multi- ethnicity Malay as lazy	Indians as Tamil speakers, Punjabi speakers, Malayalam speakers, Telugu speakers, Malay speakers, Urdu speakers Indians as English educated
			Indians as *pottu*wearing Indians as labourers Indians as alcoholics/good drinkers Indians as Black Indians as *Keling* Indian Singaporeans as different from Indian from India in Singapore Indians as favouring spicy food

These constructions will be expanded upon in the following section. Now we turn to understanding how the processes and motivations of racial identity construction influenced, and was influenced by interactions with, Others.

### Process and Motivation of Identity Constructions

While the content of the racial identities was based on colonial constructions of race, the process of identity construction and motivations behind the process was seen when participants positioned themselves alongside or against these colonial constructions of race. This positioning took place in both the contemporary constructions of their own racial identities, as well as those of Others. For example, what it means to be Malay today was juxtaposed against colonial constructions of the Malay identity, and Malay-identifying individuals would challenge these constructions by providing new constructions of the Malay identity, thereby changing the content of that representation to a more positive construction.

#### Positioning Along Colonial Constructions of Race

Participants took reference points for their own identity constructions from colonial constructions of race. In a focus group carried out in London among Malaysian participants, Chinese identity was constructed alongside the colonial construction of Chinese as “greedy” by Louisa, a self-identified Chinese Malaysian.

Extract 1:

Louisa: When I was brought up, even as a Chinese, I’m not that traditional. I don’t speak Mandarin. I don’t do all of the tradition things at home.

First author: Do you speak any dialects?

Louisa: No. My parents do. We’re not really raised in that sense. Never really thought about…

First author: So you didn’t think about what it means to be Chinese…

Louisa: I think it’s based on a lot of stereotypes, so that’s how I like picked up on

First author: OK. Who created these stereotypes?

Louisa: In school basically, when I was growing in primary school, basically like my friends were 70% Chinese. They would always label you like, Chinese people are super Kiasu [see footnote 2 above]. You know, that’s how I like started forming my own thoughts like.

While this extract seems to register the speech only of one person (and the interviewer), Louisa’s speech is intersected by the voices of other non-present speakers, showing tension between these voices, particularly from the home and school settings. Louisa constructs her Chinese identity from the position as a non-traditional Chinese, thereby distancing herself from stereotypical constructions of the Chinese identity. In constructing what she is, Louisa states what she is not, centering her construction on what are commonly thought to be symbolic practises upon which Chinese identity is constructed (such as speaking Mandarin, partaking in traditional activities at home). This example is a case of *intertextuality*, where prior representations support subsequent representations, thereby enacting a particular understanding of the Chinese identity (Elcheroth et al., 2011).

In contrast, Louisa explicitly applies the construction by Others *(“Chinese people are super Kiasu”)* in the formation of her own Chinese identity. Louisa also deflected responsibility for perpetuating this stereotypical construction by using the phrase “*they would always label you*,” removing agency from herself and directing the talk to Others in the room, instead of using the word “me” (and the “I” position) in that phrase. Other participants do not contest this hegemonic representation and move on to discuss their own experiences with the Chinese identity. The act of being “*Kiasu*” is one way of positioning oneself as Chinese, becoming a concrete enactment and social norm of the Chinese identity. By positioning herself along the colonial constructions of race, Louisa has sought to draw on common representations of the Chinese identity and changed it into an instrument that she can use in understanding what being Chinese meant to her. Drawing from SRA’s concept of world-making assumptions, the social representation of Chinese identity is transformed from one that depends on the individual being “traditional” and “speaking Mandarin.” Louisa is also transformed when she adopts this colonial label of greedy into contemporary construction of “Kiasu.”

Interestingly, Louisa’s speech highlighted here was a follow up from a group member’s response to how they constructed their identity of being Chinese. The other participant, Selena, said that she asked her parents what it meant to be Chinese, and that was how she explored her Chinese Identity. Louisa contrasts this response by saying that she *“barely explored that to be honest.”* This extract began with tension (a *dialogical knot*) that becomes resolved later in Louisa’s speech when she claims to draw upon the construction of Others in the making of her own identity. This highlights the interdependence of group members’ actions, both within the focus group and within the racial group (non-present Others) in the construction of an identity and it shows that racial identity is as much doing, as it is saying.

#### Positioning Against Colonial Constructions of Race

However, most participants positioned themselves against colonial constructions of race. Here we see two Singaporean participants, Sofia and Zara, from a focus group conducted in London using the colonial construction of Malays as “idler” to position their Malay identity.

Extract 2:

Sofia: If I may just add I think I’ve noticed all of us wanting one, we’re all Singaporean, which I’m so touched about, because as I said, I’m so much older than all of you, I grew up in a time when you were boxed, oh you’re Malay, oh you’re Chinese, oh you’re Indian, you should be doing this, oh if you’re Chinese, you cannot do art but you’re good with numbers, Indian, then you have to smell of curry, you’re very good at talking, you’re Malay, oh very lazy, oh, very stupid, but you can sing very well [group laughs]. You come from that time when segregation was the norm, and you kind of accepted that.

Zara: It was almost like character profiling.

Sofia: Yah, I think the FBI can find a new job in Singapore, don’t have to do profiling, it’s all done. They themselves, we, my time, our people, accepted that by acting in that way, I’m Malay, of course I’m very bad in Math la, I’m not very clever… of course we are poor. You know that kind of thing. You know, now what I hear from all of you, the younger ones is that ok we are all Singaporean, we are a bit of Chinese, Indian, we are a bit of Malay, we eat all the different racial food, we happily celebrate each other’s ethnic celebrations. I think we all sort of want that kind of cohesiveness, isn’t it?

Sofia introduces constructions of Malays being “*very lazy*” and “*very stupid*” to the discussion. It is interesting to see that Sofia adds the emphasis *“very”* to the racial identity that she has been categorised as (Malay), and not to other racial identities. She switches from the *I* position (“*I grew up in a time”*) to the *you* position (“*you were boxed”*) and continues to draw other focus group participants into her experience. While it may seem like a dyadic verbal interaction between Zara and Sophia, the group responds to Sofia’s introduction of colonial constructions of race. Here the group laughs, showing that they too are aware of these constructions. In this extract, tension exists not within the group, but within Sofia’s speech. She switches from the “they” position to the “I” position as she resolves this conflict in the construction. We see these representations of Malays, Chinese, and Indians as shared knowledge within the group (Elcheroth et al., 2011). In response, Zara steps in and signals her shared experience with Sofia, positioning herself as a person who grew up in the same time period. Sofia then switches positions again from “*they themselves*” to “*we, my time, our people*,” this time aligning herself with Others who accepted these constructions. However, she underpins this process with the use of “*of course”* twice, positioning herself as being outside of this construction by mocking it.

We see the multi-voiced nature in the construction of the self and ingroup (Malay) identity here in Sofia’s speech. This multi-voiced nature of the Self is considered an adaptive response to the fractured social world that we live in (Aveling and Gillespie, 2008). Sofia appeals to the participants from the younger generation by drawing differences between “*my time*,” a much younger, less aware Singapore and the current state of affairs in the country. Her construction of Malay identity here serves the purpose of illustrating a difference in the construction of racial identities from a time before. This distinction shows the evolving nature of the importance of these colonial constructions. The desire to move away from these colonial constructions comes from starting to name them as stereotypical constructions that have little relevance to what the younger generation experience – a preference for the superordinate nationality identity (“*we are all Singaporean”*) over individual racial identities. Participants bring awareness of colonial constructions of race into the group, engage with these constructions collaboratively and distance themselves from it. Stereotypes are seen as judgments of a specific category (here, race), at times different to one’s own, and which becomes a device that contains a social content (Moscovici, 2011). The stereotypes are thematised by participants, and we argue that participants are motivated to changing these stereotypes, and thus changing the content, by first acknowledging and talking about them.

Arvin and Anika, two participants from a focus group carried out in Malaysia, also constructed their Indian identities against the colonial construction of Indians as drunks. This extract highlights a problemmatic construction of Indians and shows how participants worked through this point of contention.

Extract 3:

Arvin: Uh, I’m like yah, you don’t know how much I can drink. I’m like no, I can’t drink. [Laughs]

Anika: But that’s true right, so when you say Indian, immediately you think, oh, able to drink.

Arvin: Yah, yah.

Anika: Should be able to drink the entire table. And historically, and rightly or wrongly, there’s the prejudice that Indians are, you know, labourers, working in the estates, maybe to some extent, quite edgy. And I’m not saying that this is right, I mean, this is perception, right.

Here, Arvin switches between addressing the non-present Other from his conversation outside the focus group (“*you don’t know”*), and his fellow focus group participants in the room, drawing the participants into this construction of the Indian identity. He signals his position (against the construction) reiterating his point that he does not fit into this stereotypical construction of Indians, and that it is a false construction of Indians. Anika continues to draw on the voice of the absent speaker, showing that the meta-meta knowledge (what we know about Others’ knowledge of us) of the Indian identity is instrumental in the construction of racial identities.

Tension within Arvin’s construction arises when Anika says “*But that’s true right*,” legitimising this false construction by then drawing Arvin and other focus group participants in by using “*you*” (other position). Arvin’s positive response to this *(“yah, yah”)* then leads her to ground this construction in history, further legitimising this false construction. Yet even in the validation of this construction, Anika positions herself against it by being dismissive of it with the use of the phrase “*rightly or wrongly.*” She distances herself even further when she says “*I’m not saying that this is right”* once again showing the tension between talking about this false colonial construction of Indians and the desire to reflect her own sentiment about it. In their dialogue, we see that meaning drawn from meta (and meta-meta) knowledge of Indian identity is contextual^4^ and is not simply contained in the utterance of the stereotypical construction. Both Anika and Arvin seek to change the content of this construction through meta-knowledge and, in doing so, construct the Indian identity in opposition to Others’ construction of Indians. By positioning themselves against these (negative) constructions, they create space for alternative constructions of the Indian identity. Anika embarks on this process of creating alternative constructions with the use of the word “edgy” rather than drunk, carefully co-constructing the Indian identity with Arvin.

An additional layer of complexity in identity construction takes place within this extract. Arvin, categorised by the state as *Kadazan*, a *Bumiputera* identity category, identifies with both his *Kadazan* and Indian identities. He began this extract by drawing reference to both *Kadazan* and Indian stereotypical constructions of being alcoholics. However, it is only the Indian (colonial) construction of Indian as drunk that the focus groups fixates and discusses, even when there was another member of the focus group who identified as *Bumiputera* (specifically *Bajau*). Not all negative constructions were contested in the focus groups, and this extract is a case in point.

Outgroup members frequently constructed racial identities that they did not identify with as well. In this focus group conducted in Singapore, Janet, self-identified Chinese Singaporean, constructs the Malay and Indian identity. What is interesting about the construction of the Indian identity is that Janet defers to Nadia, self-identified Indian participant in the construction of the Indian identity, positioning Nadia as a gatekeeper of the identity.

Extract 4:

Janet: Something like that, I don’t know. [Wrings hands] I don’t want any “seditious” [Airquotes]

First Author: This is not like the Sedition Act^5^

Janet: I’m totally like, digging my own… Crossing the boundaries a little bit, maybe, you know, Malays like lepak one corner, so, kind of the stereotype like, where, you know, you think Malays generally are more relaxed, they take things at a slower pace, they have different kind of culture, they are very tight-knit, something like that.… Indians, my mum keeps thinking that, Indians, they are very good speakers, as what she said that’s why we have so many doctors and lawyers [Nadia nods] from there, because they are such good speakers.

Nadia: Like…

Janet: Like they like to argue, this kind of thing. Because my Indian neighbour is, he always goes down to the void deck, he talks to a old bunch of ladies, and he every time, he’s like the group’s mover, you know. So he’s like, always travelling with his things someplace. But he’s quite unique himself, he can speak Teochew…

Nadia: Yes. [Nods]

Janet begins this dialogue in an apologetic tone, aware that she is bringing up some controversial issues embedded in the construction of the Malay and Indian identities. She highlights this tension within her own dialogue when she switches from one position to another (“*I’m totally like”; “you know you think”; “my mum”*). She brings in her mother’s perspective (absent speaker) into the discussion as a powerful point of view in establishing the stereotypical constructions of which she is aware. The invoking of this stereotype clearly made Janet uncomfortable, and we can see how she resisted the construction of Indians as “*good speakers*” by creating a distance when referring to her mother’s views instead of hers. This *dialogical knot* illustrates the conciliatory approach taken by Janet in discussing such essentialised constructions of different racial categories in Singapore. In this particular focus group, views about minority were discussed tentatively, and the other participants frequently looked to Nadia, the only racial minority member, for approval and acceptance. That Nadia did not question this positive construction of Indians and that the conversation progresses smoothly shows not only how Janet is able to draw on common references in the construction of the Indian identity, but also that belief in this construction allows Janet to elicit the support of her focus group members. Her mobilisation of this stereotypical construction, though tentative at first, gives her clues about the manner with which the conversation should unfold so as to elicit support from other people in that group setting.

That Nadia herself is a practising lawyer, adds a dimension of credibility to Janet’s construction of Indians as *“doctors and lawyers.”* From Janet’s perspective, it also becomes a self-fulfilling prophecy. This is of course a positive construction of the Indian identity, and one that does not fall into the colonial construction of Indians. Janet’s position is vastly different from the colonial construction of Indians as drunk labourers, demonstrating a crucial development in identity construction. It is also perhaps telling that this construction of Indians is elaborated on in this dialogue, rather than the colonial construction of Malays that Janet starts the dialogue with, showing Janet’s positioning of herself as being against colonial constructions of race. In the space created by positioning herself against the colonial construction of Indians, Janet then puts forth a (positive) contemporary construction of the Indian identity. She has drawn on shared knowledge of the negative construction of Indians to change content of the Indian identity.

It is noteworthy that outgroup constructions of racial identities that resembled colonial constructions were often framed in a softer manner when they were speaking from their own voices as mentioned earlier. The colonial construction of “Malays as idlers” is constructed as “Malays are relaxed and not industrious” by Chinese identifying individuals. Janet’s speech shows a hesitation to use a overtly negative stereotype on another group of individuals. Even when Indian identifying individuals constructed “Malays as lazy and entitled” as seen in Anika’s speech below, she is seen to reflect the voice of the non-present Other.

Extract 5:

Anika: “And it’s also the butt of jokes. So you know, Indians and then Chinese are very hardworking, very entrepreneurial, very crude, very sharp, kiasu, then Malay is very lazy, entitled, because with all the bumiputera um, you know, selection policies, and um, affirmative action policies that benefit them. So rightly or wrongly, and this is very specific to Malaysia and maybe to a lesser extent, Singapore.”

We see that Anika draws on shared understandings of jokes that target specific racial groups in Malaysia and Singapore to discuss this negative construction of Malays. She does not claim to feel this way. In other words, she does not speak from her own voice, and directs the reader to what she presents as opinions that are present in the society at large. She thus introduces these negative constructions into the discussion in a manner that she hopes will not raise tension within the focus group, however, we note the tension in her speech. She seeks to clarify the construction by bringing in examples of social policies that are beneficial for Malays in Malaysia and also marks her speech with a caveat by stating *“rightly or wrongly.”*

Colonial constructions of Malay, Chinese and Indian identities are embedded in the contemporary constructions of the same identities. Yet, what needs to be noted is that some colonial constructions were actively positioned against, such as the Indian and Malay identity, while the Chinese identity was positioned alongside the colonial construction. Indian and Malay colonial constructions were seen as wholly negative and thus participants contested them and distanced themselves from these constructions, creating new constructions juxtaposed against the old. However, the colonial Chinese construction is associated with more positive qualities such as being enterprising and being successful as a result of being “Kiasu” (see Table 4 where all three racial groups constructed Chinese as enterprising). This positive perception could be a reason for the endurance of the colonial construction of Chinese identity.

### Limitations

Before we summarise our findings, we would like to address potential for future research. A limitation of this study was that all participants had at least GCSE ‘O’ level (or equivalent) education, and were fluent in English. While this is largely representative of the English speaking Singaporean population, it does not represent much of Malaysia, where Malay is the lingua franca. Perhaps individuals who are less fluent and comfortable communicating in English would have different constructions of the races present in their countries. Malaysian participants were also only sampled from Kuala Lumpur, as we only had resources to conduct the focus group discussions for Malaysia in that location. We expect that discussions around racial identity would be different if the study was conducted in more rural parts of West Malaysia or in East Malaysia. While in many ways meaningful in understanding the construction of racial identity in Malaysia and Singapore, we have limited our discussion on the interconnected nature of racial and national identity in this paper, which we address elsewhere (Reddy, 2018) because of our focus on developing a deeper understanding of the commonalities in the psychology of racial identity construction among Malaysians and Singaporeans. We encourage futher research understanding the complexities of hyphenated identities that encompass racial/ethnic and national identities.

## Conclusion

Empirically, this paper presents an often overlooked aspect of the context that contributes to racial identity construction - that of the (colonial) history. This oversight might be because much of social and cultural psychology today focuses on the immediate social context within which identities are constructed and negotiated. There is an increasing focus on the part of psychologists to address this oversight by engaging with coloniality and what that means for the psychology of individuals today. This focus is poignantly seen in recent work such as the special issues of the International Journal of Intercultural Relations edited by Bobowik et al. (2018) and the Journal of Social and Political Psychology edited by Adams et al. (2015), amongst others. This paper contributes to this growing body of research that seeks to expose the historical roots of how identities and identity categories came to exist in their current state. Specifically, this study reinforces Okazaki et al. (2008) call for more psychological research to be conducted to understand how major geopolitical events, such as colonisation, influence people’s lives.

A strength of this paper that has allowed us to explore the influence of colonial history in racial identity construction is its qualitative methodology. While quantitative analysis continues to provide observations that further our understanding of social representations and, by extension identities (Doise et al., 1993), qualitative methods and analysis have provided us with the opportunity for unique insights that we have gleaned about the influence of colonial symbols in contemporary racial identity construction. The methodology chosen for this study allowed us to elucidate the ways identities are dynamically disrupted, representational conflicts are resolved and identities are reconstructed in a mutual constitution of social and cultural worlds. Specifically, the method of data collection, the type of questions asked, and the type of analysis utilised in this paper were especially useful in documenting the mutual constitution of culture (seen through colonial representations of race) and psyche (seen through one’s personal experience of identity). A key concern in the research process was to understand how racial identities were constructed and re-constructed in the presence of Others who were physically present, in addition to those who were implied and imagined. Specifically, an understanding of the dialogical interplay of the Self and Other, as well as the role of the social context as outlined in Reicher (2004) was needed at this step for the study. Focus groups were used as valuable resources for data collection as they move beyond “essentially individualistic framework” (Puddifoot, 1995, p. 364) and examine the inter-subjective level of social identities on dialogicality, that is, the “capacity to conceive, create and communicate about social realities in terms of Otherness” (Marková, 2003, p. 91). Thus, the choice of focus group data facilitated a deeper understanding of the connectedness between individuals in the racial identity construction process. Furthermore, the use of open-ended questions supplemented by relevant prompts in small group discussions faciliated the probing of complex ideological tools that participants drew upon in their construction, that perhaps would have been more challenging to elict in a quantitative survey or experimental set-up. In addition, we drew on one of the core functions of SRT that connects ideological systems in social and political life (Jovchelovitch, 2007) for our DA. That is to say that social representations are viewed as ideological tools that can facilitate the exploration of inequality and stigma (Howarth, 2009). Therefore, DA allowed us to examine the content, process and motivations of identity construction.

A key contribution of the paper is an expansion of the psychological conceptualisation of the socio-political context. Rather than drawing reference from the immediate perceptual context or specific moments in time, we found that the psychological traces of colonialism still echo in the self- presentation, construction and negotiation of racial identities of individuals from Malaysia and Singapore. Participants, regardless of differences in socio-political contexts, as characterised by different geographical locations, similarly engaged with colonial constructions of race in constructing contemporary Malay, Indian, and Chinese racial identities. Importantly, the colonial representations served the purpose of a providing a reference point, a way with which people organise and view their social worlds. We stress that the core of these constructions is based on colonial representations of race. Both the idea of categorising people according to discrete, “racial differences” and the contents of what these racial categories mean are rooted in colonial constructions, and have endured till today. These findings echo those of other scholars who found that the colonial past is relevant to one’s social identity, and embedded in present life, if they have been colonised (David and Nadal, 2013; Licata et al., 2018). Coloniality is not simply restricted to the distant past but is very much ongoing when seen in the light of intercultural (and intergroup) relations (Adams et al., 2018). Because identity is also located culturally and historically (Hammack, 2008), the socio-political context needs to more explicitly include historical and cultural elements. We extend Hammack’s point by defining culture to include the post-colonial. Postcoloniality means that cultural legacies of colonial symbols still influence the psychology of contemporary society (Patke, 2005) and that culture can be conceptualised beyond fixed national and cultural boundaries (Bhatia and Ram, 2001). Indeed, what we show is that the psychology of racial construction is embedded in a space that surpasses fixed geographical boundaries of nationhood and culture.

Therefore, we argue that the conceptualisation of the socio-political context should include the ideological context of colonialism and post-coloniality. In this sense, socio-political contexts are not just demarcated by geographical locations and, by extension, contemporary political ideologies, but also can be rooted in historical experiences that create a powerful ideological context that crosses geographical boundaries. When context is examined through lens of colonial history, we see that the differences in geographical location (United Kingdom, Malaysia, and Singapore) and specific moments in time (different focus groups) are not the only frames within which identity construction takes place. Rather, we posit that the socio-political context can also be understood as everyday engagments with colonial symbols that transcend geographical locations and moments in time. We call for researchers to expand the conceptualising of context beyond that which is usually studied. We argue that psychological understanding of context should not only be embedded in spaces and specific moments in time, but also in the embodied mind that has experienced colonialism and continues to perpetuate colonial thinking. Colonialism is not a thing of the past. It continues to influence our contemporary ways of being not only in physical ways (such as colonial architecture, colonial social policies) but also in its omnipresence in our minds and bodies. In essence, context is time (and history) moving through embodied minds, as much minded bodies move through spaces and time.

Theoretically, we have extended the SRA concepts of meta and shared knowledge (Elcheroth et al., 2011) in the application of the study of racial identities. Notably, extracts 2 and 4 show how participants draw on meta and shared knowledge to change the contents of the representations of Malay and Indian racial identities from a colonial construction to a contemporary construction. In doing so, they also frame their identities within this changed content. These enduring colonial representations provided the foundation for the change in the construction of racial identities among our participants. The use of these colonial representations did not mean that participants accepted them wholly. Participants challenged and contested these colonial constructions of race when constructing their racial identities today. The defining property of a social representation is not that it should be shared in the same way by everyone who uses such a representation. Rather, the internal structure of the representation and the extent to which it is dispersed within a group or social category will depend on the functions that it serves. As seen from extracts 1 and 3, participants’ knowledge of a representation of the Chinese and Indian identities allowed them to form their constructions of their own racial identities. This finding is significant as it contributes to a fuller understanding of the SRA paradigm by showing how individuals use meta and shared knowledge to change their identities. This aspect of identity change is not addressed in the current SRA framework. Therefore, this paper proposes a dynamic view of SRA in action, showing how social representations are not static descriptions of the reflections of society but rather are re-presentations of the social world that have the potential for change within society. By re-presenting their identities, we posit that participants are engaging in social change. This presents possibilities for a new reality, therefore connecting the four facets of SRA (meta-knowledge, shared knowledge, enacted communication, and world making assumptions) in the study of racial identity construction.

Lastly, we see that colonial representations of race are central to constructions of different racial identities in Singapore and Malaysia by *both ingroup and outgroup members*. Racial identity constructions are not limited to minority group members, as shown in research discussed above (e.g., Verkuyten, 1997; Shih et al., 2007). All participants used the process of identity construction to understand how Others position them, and how they should position themselves to Others. Thus, racial identity takes on a strategic role, informing Singaporean and Malaysian individuals of Chinese, Malay, and Indian racial identity about how to interact with one another in group settings. We show that because racial identity construction is inherently relational, participants engage in them beyond the motivation of increasing positive self-esteem. Participants use these constructions to connect with one another, as seen in extract 1 and 2, and to ascertain how to interact with one another, as seen in extract 4. Participants also manage tension when introducing negative constructions into group discussions, as seen in extract 5, and here identity construction is also a diplomatic exercise undertaken in group settings.

Even so, participants express discomfort when engaging with these representations, and distance themselves from the negative aspects of these constructions. There is an awareness that the racial categories, and associated colonial constructions, are insulting and inappropriate. Participants are aware that these colonial constructions of race are limiting and do not necessarily represent their own views on race and racial categorisation in these countries. Nonetheless, they engage with them because it gives them not only a common understanding of racial identities, but also a way to interact with one another in group settings, which is telling of the enduring, yet contested, nature of these representations and the power that the ideological context of colonialism presents in the embodied minds of individuals.

At a broader level, the paper contributes to ongoing discussions in the field of cultural psychology with regards to deepening the field’s understanding of culture. Defining culture is considered a futile attempt given its widespread useage across academic and non-academic contexts, and starkly different conceptualisations, as concluded by Jahoda (2012). But a nuanced understanding of culture is necessary to continue our study on the psychology of the human condition. Not only will the study of intergroup relations benefit from a more explicit inclusion of culture, the study of culture will also stand to gain from an exploration of the psychology of intergroup relations. We argue that because the Self and culture are not mutually exclusive (Hermans, 2001), and the Other is part of the Self (Bakhtin, 1981), the study of culture itself should also be one that addresses the connections between the Self and the Other. A cultural psychological perspective is one that connects “embodied personal dispositions and the cultural-ecological structures that continually tune those dispositions” (Adams and Kurtiş, 2012, p. 193) and one that studies the “intentional worlds” of individuals (Shweder, 1990, p. 3). Culture has also been conceptualised as a “collective programming of the mind” that distinguishes one group of individuals from the other (Hofstede, 1984, p. 21), highlighting that the study of intergroup differences and intergroup relations needs to be embedded in a cultural psychology approach. Therefore, we suggest that the study of culture for psychologists is one that connects (1) individual characteristics, (2) the many social worlds of the individual, (3) culture as an institutional structure and (4) intergroup relations. To this end, this paper brings together these four aspects - specifically the co-constructions of racial identities by individuals belonging to different groups within a conceptualisation of culture that includes the colonial institution. Historical events lead to lasting cultural legacies and political ideologies in the constructions of racial identities by different groups in society, showing how the past still has a place in contemporary psychologies, especially in the cultural and social psychological imagination of race.

## Ethics Statement

This study was carried out in accordance with the recommendations of British Psychological Society ethics guidelines by the Chair of the Department of Psychological and Behavioural Sciences ethics committee, as well as Research Degrees subcommittee of the London School of Economics and Political Science, United Kingdom, with written informed consent from all subjects. All subjects gave written informed consent in accordance with the Declaration of Helsinki. The protocol was approved by the Chair of the Department of Psychological and Behavioural Sciences ethics committee.

## Author Contributions

GR constructed the study, carried out the data collection, analysis, and wrote the manuscript. IG provided key supervisory assistance and edited the manuscript. Both authors contributed to manuscript revision read and approved the submitted version.

## Conflict of Interest Statement

The authors declare that the research was conducted in the absence of any commercial or financial relationships that could be construed as a potential conflict of interest.

## Footnotes

1 Self is capitalised to distinguish from a reference by a subject to the same subject. Other is capitalised to differentiate from the interpersonal other.

2 It must be noted that DA does not require an enumeration of themes or codes. This type of analysis is better captured in a Content Analysis (see Bauer, 2000; Hsieh and Shannon, 2005). Rather, DA focuses on locating these *dialogical knots* during the construction of an identity. Aveling and Gillespie (2008) provide a good account of how DA can be used to understand identity constructions.

3 Kiasu is a Hokkien (Chinese) term and a cultural concept for a negative form of competition that is said to promote selfishness and stem from greed (Ho et al., 1998).

4 Context is conceptualised here as both a historical context (period of colonisation) as well as a geographical location (Malaysia).

5 To date, public discussions regarding race, language or religion are considered to be taboo and discussions are censored by the state and citizens alike (George, 2000).

## Appendix A Interview schedule.

**Table:**

Introduction	
Thank you for agreeing to take part in this research study.(Author introduction)I hope to get as much information as possible so please share as many details and opinions. There are no right or wrong answers. I am interested in your opinions. The discussion will be video recorded so that it can be transcribed later. This will help me understand better what everyone is saying. No names or personal identifiers will be used at any stage of the analysis. All information will be kept confidential and will be used for research purposes only. Are there any questions at this stage? Ok, before we begin, could you share with me why you agreed to take part in this interview?	
Main Discussion points	Prompts
Ok let’s go around the group and introduce ourselves. We can state our names (or names that you would like to be called in this group), pronouns that you would use to call yourself, and the item that you have brought along that encapsulates your ethnic identity.	
What racial category was used to describe you when you were born?	
What are the ways you explored your Malay/Indian/Chinese Identity?	What makes you Malay/Indian/Chinese? Language? Food? What does the Malay/Indian/Chinese culture mean to you?
How does your idea of your ethnicity/race differ from others in your ethnic/racial group?	How representative of your ethnic group are you? Do you find it easy to be a member of your ethnic group?
When you see another person of your ethnicity/race, what language do you speak to them in?	How do you decide if someone you have never met is of your ethnicity/race?
What do you think of interethnic marriage?	Do you have friends/other family members (not from your immediate family) who are mixed?
Suitable Vignette from list (attached below)	What do you think about this statement?
Malaysia/Singapore is seen as a multiracial country. What makes it multi racial?	What are some ways that Malaysia/Singapore is multiracial? What is the importance of Malaysia being multi racial?
How similar is being Malay/Indian/Chinese in Malaysia/Singapore and London?	
We are now at the end of our discussion and I would like to get some feedback from you. Considering all the issues discussed this afternoon, which do you feel are the most important issues discussed?	Have we missed out any important issue?

## Vignette List

**Table:**

Topic	Vignette
Institutional Racism experienced when multiracial	And such an experience happened when I was 16, so I went up to visit my Chinese friend and Indian friend in a restaurant. So it’s fasting month and I was eating and enjoying myself with my friends and talking. There were these authorities from Jakim, the Islam Council, and they were conducting raids in restaurants to catch any Muslims or Malays eating food and not doing their fasting and stuff. So I was eating and suddenly there was someone slapping my shoulder very hard and it caused a lot of pain and I turned around and there was this officer. And I said, ‘no, I’m not, I’m a Chinese mixed Indian,’ but they don’t believe, and to the extent that I had to bring out my identity, my IC and then he refused to look at the IC. They just grabbed me and put me in the car. And then went to the police station and I sit there for hours, called up my parents, my mother basically but my mother can’t come because she’s working. So they phoned up my aunt and when they called up, they were so surprised why is he Chinese and then I said, ‘I only asked you to look at my IC but you did not, you refused.’
Indian and Chinese experience similar discrimination	I feel that the Indian community and the Chinese community are somewhat playing on a somewhat similar playing field. I think they both face discrimination. They both face similar issues in their lives. They go through about the same level of racism as well in other countries and also within their own country as well. So in many ways, they do have a lot of similarities that they’ve not actually acknowledged that they do.
Acceptance of multiracial individual by one racial group	First Author: How did your friends see you? Vino: The majority of my very close childhood friends saw me as Chinese sometimes. Another Chinese girl who was just bigger, a little darker, had an Indian father and an Indian name. Apart from that, she was very much like… I was very much like them and they accepted me. I started picking up a lot of Chinese dialects. I ate a lot of Chinese food. I wanted to do all the fun activities with my friends. And apart from that, everybody would just call me Vino you know.
Choice of one racial identity over the other	My mum is a bit antagonistic toward my dad so she’s always telling me, don’t be like your dad, he’s lazy because we’ve got this stereotype about Malays. So she’s always like, don’t be like your dad, don’t be lazy, don’t be this, don’t be that and after a while, those stereotypes got in my mind and I started identifying myself as a Chinese more.

## References

[B1] Adams G. Dobles I. Gómez L. H. Kurtiş T. (Molina) L. E. eds) (2015). Decolonizing psychological science: introduction to the special thematic section [Special issue]. *J. Soc. Polit. Psychol.* 3 213–238.

[B2] Adams G. Estrada-Villalta S. Ordóñez L. H. G. (2018). The modernity/coloniality of being: hegemonic psychology as intercultural relations. *Int. J. Intercult. Relat.* 62 13–22. 10.1016/j.ijintrel.2017.06.006

[B3] Adams G. Kurtiş T. (2012). “Context in person, person in context: a cultural psychology approach to social-personality psychology,” in *The Oxford Handbook of Personality and Social Psychology*, Deaux K. Snyder M. (Oxford: Oxford University Press), 182–208.

[B4] Adams G. Salter P. S. (2007). Health psychology in African settings: a cultural-psychological analysis. *J. Health Psychol.* 12 539–551. 10.1177/1359105307076240 17440003

[B5] Agence France-Presse (2015). Malaysia Strengthens Sedition Law in a “Black day” for Free Speech. *The Guardian.* Available at: https://www.theguardian.com/world/2015/apr/10/malaysia-strengthens-sedition-law-in-a-black-day-for-free-speech (accessed September 20, 2017).

[B6] Andreouli E. Howarth C. (2013). National identity, citizenship and immigration: putting identity in context. *J. Theory Soc. Behav.* 43 361–382. 10.1111/j.1468-5914.2012.00501.x

[B7] Aveling E.-L. Gillespie A. (2008). Negotiating multiplicity: adaptive asymmetries within second-generation Turks “society of mind.” *J. Construct. Psychol.* 21 200–222. 10.1080/10720530802070635

[B8] Aveling E. L. Gillespie A. Cornish F. (2014). A qualitative method for analysing multivoicedness. *Qual. Res.* 15 22–28. 26664292 10.1177/1468794114557991 PMC4641508

[B9] Bakhtin M. (1981). *The Dialogic Imagination: Four Essays*, ed. Holquist. M. Austin, TX: University of Texas Press.

[B10] Bauer M. W. (2000). “Classical content analysis: a review,” in *Qualitative Researching with Text, Image and Sound: A Practical Handbook*, eds Bauer M. W. Gaskell G. (London: Sage), 131–151.

[B11] Bhatia S. Ram A. (2001). Rethinking ‘acculturation’ in relation to diasporic cultures and postcolonial identities. *Hum. Dev.* 44 1–18. 10.1159/000057036

[B12] Bianchi F. Zea M. C. Belgrave F. Z. Echeverry J. J. (2002). Racial identity and self-esteem among black Brazilian men: race matters in Brazil too! *Cult. Divers. Ethnic Minor. Psychol.* 8 157–169. 10.1037/1099-9809.8.2.157 11987592

[B13] Billig M. (1993). Studying nationalism as everyday ideology. *Papers Soc. Represent.* 2 40–43.

[B14] Billig M. (1995). *Banal Nationalism.* London: Sage Publications.

[B15] Bobowik M. Valentim J. P. Licata L. (2018). Introduction to the special issue: colonial past and intercultural relations [Special issue]. *Int. J. Intercult. Relat.* 62 1–12. 10.1016/j.ijintrel.2017.10.003

[B16] Bowker G. C. Star S. L. (2000). *Sorting Things Out: Classification and Its Consequences.* Cambridge, MA: MIT Press.

[B17] Brah A. (1996). *Cartographies of Diaspora: Contesting Identities.* London: Routledge.

[B18] Chua B. H. (2005). When difference becomes an instrument of social regulation. *Ethnicities* 5 418–421. 10.1177/146879680500500310 25199924

[B19] Clark K. B. Clark M. (1947). “Racial identification and preference in Negro children,” in *Readings in Social Psychology*, eds Newcomb T. M. Hartley E. L (Oxford: Holt).

[B20] Cornish F. (2004). Making ‘context’ concrete?: a dialogical approach to the society-health relation the society-health relation. *J. Health Psychol.* 9 281–294. 10.1177/1359105304040894 15018728

[B21] David E. J. R. Nadal K. L. (2013). The colonial context of Filipino American Immigrants’ psychological experiences. *Cultur. Divers. Ethnic. Minor. Psychol.* 19 298–309. 10.1037/a0032903 23875854

[B22] Deaux K. (2006). *To be an Immigrant.* New York, NY: Russell Sage Foundation.

[B23] Desrosières A. (1990). “How to make things which hold together: social science, statistics and the state,” in *Discourses on Society*, eds Wagner P. Wittrock B. Whitle R. (Dordrecht: Kluwer Academic Publishers), 195–218.

[B24] Doise W. Clemence A. Lonrenzi-Cioldi F. (1993). *The Quantitative Analysis of Social Representations.* Hemel Hempstead: Harvester Wheatsheaf.

[B25] Durrheim K. Dixon J. (2005). Studying talk and embodied practices: toward a psychology of materiality of “race relations.” *J. Commun. Appl. Soc. Psychol.* 15 446–460. 10.1002/casp.839

[B26] Durrheim K. Dixon J. (2010). Racial contact and change in South Africa. *J. Soc. Issues* 66 273–288. 10.1111/j.1540-4560.2010.01645.x

[B27] Duveen G. (2001). “Representations, identities, resistance,” in *Representations of the Social: Bridging Theoretical Tradition*, eds Deaux K. Philog G.ène (Oxford: Blackwell).

[B28] Edwards D. (1998). “The relevant thing about her: social identity categories in use,” in *Identities in Talk*, eds Antaki C. Widdicombe S. (London: Sage), 15–33.

[B29] Elcheroth G. Doise W. Reicher S. (2011). On the knowledge of politics and the politics of knowledge: how a social representations approach helps us rethink the subject of political psychology. *Polit. Psychol.* 32 729–758. 10.1111/j.1467-9221.2011.00834.x

[B30] Fanon F. (1967). *The Wretched of the Earth. [1961].* (trans. Constance Farrington. Reprint.) New York, NY: Grove.

[B31] Farr R. M. Trutkowski C. Holzl E. (1996). Public opinion, group discussion and theory of social representations. *Res. Pap. Psychol.* 9602.

[B32] Gabriel S. P. (2015). The meaning of race in Malaysia: colonial, post-colonial and possible new conjunctures. *Ethnicities* 15 782–809. 10.1177/1468796815570347

[B33] George C. (2000). *Singapore: The Air-Conditioned Nation: Essays on the Politics of Comfort and Control.* Singapore: Landmark Books.

[B34] Giddens A. (1987). *Social Theory and Modern Sociology.* Cambridge: Polity Press.

[B35] Gillespie A. Cornish F. (2010). Intersubjectivity: towards a dialogical analysis. *J. Theory Soc. Behav.* 40 19–46. 10.1111/j.1468-5914.2009.00419.x

[B36] Gillespie A. Howarth C. S. Cornish F. (2012). Four problems for researchers using social categories. *Cult. Psychol.* 18 391–402. 10.1177/1354067X12446236

[B37] Hammack P. (2008). Narrative and the cultural psychology of identity. *Pers. Soc. Psychol. Rev.* 12 222–247. 10.1177/1088868308316892 18469303

[B38] Harrison C. W. (1929). *Some Notes on the Government Services in British Malaya.* London: Malayan Information Agency.

[B39] Harrison F. V. (1995). Annual reviews the persistent power of “race” in the cultural and political economy of racism. *Source: Annu. Rev. Anthropol.* 24 47–74.

[B40] Hashim R. Tan C. (2009). A hyphenated identity: fostering national unity through education in Malaysia and Singapore. *Citizenship Teach. Learn.* 5 46–59.

[B41] Haslam S. A. Turner J. C. Oakes P. J. McGarty C. Hayes B. K. (1992). Context-dependent variation in social stereotyping 1: the effects of intergroup relations as mediated by social change and frame of reference. *Eur. J. Soc. Psychol.* 22 3–20. 10.1002/ejsp.2420220104

[B42] Hermans H. (2001). The dialogical self: toward a theory of personal and cultural positioning. *Cult. Psychol.* 7 243–281. 10.1177/1354067X0173001

[B43] Hilton B. T. (2011). Frantz fanon and colonialism: a psychology of oppression. *J. Sci. Psychol.* 12 45–59.

[B44] Hirschman C. (1986). The making of race in colonial Malaya: political economy and racial ideology. *Sociol. Forum* 1 330–361. 10.1007/BF01115742

[B45] Ho J. T. S. Ang C. E. Loh J. Ng I. (1998). A preliminary study of kiasu behaviour - is it unique to Singapore? *J. Manger. Psychol.* 13 359–370. 10.1108/02683949810220015

[B46] Hofstede G. (1984). Cultural dimensions in management and planning. *Asia Pacific J. Manage.* 1 81–99. 10.1007/BF01733682

[B47] Hopkins N. Kahani-Hopkins V. (2004). Identity construction and British Muslims’ political activity: beyond rational actor theory. *Br. J. Soc. Psychol.* 43 339–356. 10.1348/0144666042037935 15479534

[B48] Hopkins N. Reicher S. (2011). Identity, culture and contestation: social identity as cross-cultural theory. *Psychol. Stud.* 56 36–43. 10.1007/s12646-011-0068-z

[B49] Howarth C. (2002). Identity in whose eyes? The role of representations in identity construction. *J. Theory Soc. Behav.* 32 145–162. 10.1111/1468-5914.00181

[B50] Howarth C. (2009). “I hope we won’t have to understand racism one day”: researching or reproducing “race” in social psychological research? *Br. J. Soc. Psychol.* 48 407–426. 10.1348/014466608X360727 18817595

[B51] Howarth C. Wagner W. Magnusson N. Sammut G. (2013). “It’s only other people who make me feel black”: acculturation, identity, and agency in a multicultural community. *Polit. Psychol.* 35 81–95. 10.1111/pops.12020

[B52] Hsieh H. F. Shannon S. E. (2005). Three approaches to qualitative content analysis. *Qual. Health Res.* 15 1277–1288. 10.1177/1049732305276687 16204405

[B53] Hua W. Y. (1983). *Class and Communalism in Malaysia: Politics in a Dependent Capitalist State.* London: Zed Books.

[B54] Jahoda G. (2012). Critical reflections on some recent definitions of “culture”. *Cult. Psychol.* 18 289–303. 10.1177/1354067X12446229

[B55] Jovchelovitch S. (2007). *Knowledge in Context: Representations, Community and Culture.* London: Routledge 10.4324/9780203968895

[B56] Kahani-Hopkins V. Hopkins N. (2002). “Representing” British Muslims: the strategic dimension to identity construction. *Ethnic Rac. Stud.* 25 288–309. 10.1080/01419870120109494

[B57] Lauri M. A. (2009). Metaphors of organ donation, social representations of the body and the opt-out system. *Br. J. Health Psychol.* 14 647–666. 10.1348/135910708X397160 19159505

[B58] Licata L. Khan S. S. Lastrego S. Cabecinhas R. Valentim J. P. Liu J. H. (2018). Social representations of colonialism in Africa and in Europe: structure and relevance for contemporary intergroup relations. *Int. J. Intercult. Relat.* 62 68–79. 10.1016/j.ijintrel.2017.05.004

[B59] Luke A. Carrington V. (2002). “Globalisation, literacy curriculum practice,” in *Raising Standards in Literacy*, eds Fisher R. Lewis M. Brooks G. (London: Routledge), 231–250. 10.4324/9780203166222_chapter_16

[B60] Mama A. (1995). *Beyond the Masks: Race, Gender and Subjectivity.* London: Routledge.

[B61] Mamdani M. (2012). *Define and Rule: Native as Political Identity.* Cambridge: Harvard University Press 10.4159/harvard.9780674067356

[B62] Marková I. (2003). *Dialogicality and Social Representations. The dynamics of mind.* Cambridge, UK Cambridge University Press.

[B63] Markus H. R. (2010). “Who am I? Race, ethnicity, and identity,” in *Doing Race: 21 Essays for the 21st Century*, eds Markus H. Rose Moya P.M. L. (New York, NY: W. W. N. & Co), 359–389.

[B64] Markus H. R. Kitayama S. (1991). Culture and the self: implications for cognition, emotion, and motivation. *Psychol. Rev.* 98 224–253. 10.1037/0033-295X.98.2.224

[B65] Moscovici S. (1984). “The phenomenon of social representations,” in *Social Representations*, eds Moscovici S. Farr R. (Cambridge: Cambridge University Press), 3–69.

[B66] Moscovici S. (1988). Notes towards a description of Social Representations. *Eur. J. Soc. Psychol.* 18 211–250. 10.1002/ejsp.2420180303

[B67] Moscovici S. (2011). An essay on social representations and ethnic minorities. *Soc. Sci. Informat.* 50 442–461. 10.1177/0539018411411027

[B68] Mutalib H. (2012). Singapore’s ethnic relations’ scorecard. *J. Dev. Soc.* 28 31–55. 10.1177/0169796X1102800102

[B69] Noor N. M. Leong C.-H. (2013). Multiculturalism in Malaysia and Singapore: contesting models. *Int. J. Intercult. Relat.* 37 714–726. 10.1016/j.ijintrel.2013.09.009

[B70] Office of National Statistics (2013). Available at: http://webarchive.nationalarchives.gov.uk/20160105160709/http://www.ons.gov.uk/ons/dcp171776_346219.pdf (accessed September 20, 2017).

[B71] Okazaki S. David E. J. R. Abelmann N. (2008). Colonialism and psychology of culture. *Soc. Personal. Psychol. Compass* 2 90–106. 10.1111/j.1751-9004.2007.00046.x

[B72] Paranjpe A. C. Ho D. Y. F. Rieber R. W. (1988). *Asian Contributions to Psychology.* New York, NY: Praeger.

[B73] Park B. Judd C. M. Ryan C. S. (1991). Social categorization and the representation of variability information. *Eur. Rev. Soc. Psychol.* 2 211–245. 10.1080/14792779143000079

[B74] Patke R. S. (2005). Postcolonial cultures. *Theory Cult. Soc.* 23 369–372. 10.1177/026327640602300267

[B75] Puddifoot J. (1995). Dimensions of community identity. *J. Commun. Appl. Soc. Psychol.* 5 357–370. 10.1002/casp.2450050507

[B76] Ramiah A. Al Schmid K. Hewstone M. Floe C. (2015). Why are all the white (Asian) kids sitting together in the cafeteria? Resegregation and the role of intergroup attributions and norms. *Br. J. Soc. Psychol.* 54 100–124. 10.1111/bjso.12064 24597949

[B77] Reddy G. (2016). “Race rules in Singapore,” in *Singapore: Negotiating State and Society, 1965-2015*, eds Lim J. Lee T. (London: Routledge), 54–75.

[B78] Reddy G. (2018). *The Influence of Socio-Political Context on Racial Identity: Strategic Identity Construction Among Malaysians and Singaporeans.* PhD thesis, The London School of Economics and Political Science, London.

[B79] Reddy G. Selvanathan H. P. ((in press). “Mixed in malaysia: categories, classification and campur in contemporary everyday life,” in *Measuring Mixedness: Counting* and Classifying Mixed Race and Mixed Ethnic Identity Around the World, eds Rocha Z. Aspinall P. (Basingstoke: Palgrave Macmillan).

[B80] Reicher S. (2004). The context of social identity: domination, resistance, and Change. *Polit. Psychol.* 25 921–945. 10.1111/j.1467-9221.2004.00403.x

[B81] Rocha Z. L. (2014). ‘Stretching out the categories’: Chinese/European narratives of mixedness, belonging and home in Singapore. *Ethnicities* 14 279–302. 10.1177/1468796813505554

[B82] Rosch E. (1978). “Principles of categorisation,” in *Cognition and Categorization*, eds Rosch E. Lloyd B. B. (Hillsdale, NJ: Lawrence Erlbaum Associates), 27–48.

[B83] Said E. (1979). *Orientalism.* New York, NY: Vintage.

[B84] Sellers R. M. Caldwell C. H. Schmeelk-Cone K. H. Zimmerman M. A. (2003). Racial identity, racial discrimination, perceived stress, and psychological distress among African American young adults. *J. Health Soc. Behav.* 44 302–317. 10.2307/1519781 14582310

[B85] Sellers R. M. Rowley S. A. Chavous T. M. Shelton J. N. Smith M. A. (1997). Multidimensional inventory of black identity: a preliminary investigation of reliability and constuct validity. *J. Personal. Soc. Psychol.* 73:805 10.1037/0022-3514.73.4.805

[B86] Shih M. Bonam C. Sanchez D. Peck C. (2007). The social construction of race: biracial identity and susceptibility to stereotypes. *Cult. Divers. Ethnic Minor. Psychol.* 13 125–133. 10.1037/1099-9809.13.2.125 17500601

[B87] Shope J. H. (2006). “You can’t cross a river without getting wet”: a feminist standpoint on the dilemmas of cross-cultural research. *Qual. Inq.* 12 163–184. 10.1177/1077800405282792

[B88] Shweder R. A. (1990). “Cultural psychology: what is it?,” in *Cultural Psychology: Essays on Comparative Human Development*, eds Stigler J. Shweder R. Herdt G. (Cambridge: Cambridge University Press), 1–46.

[B89] Sinha D. (1981). “Social psychology in India: a historical perspective,” in *Perspectives on Experimental Social Psychology in India*, ed. Pandey J. (New Delhi: Concept), 3–17.

[B90] Staerklé C. Clémence A. Spini D. (2011). Social representations: a normative and dynamic intergroup approach. *Polit. Psychol.* 32 759–768. 10.1111/j.1467-9221.2011.00839.x

[B91] Stevenson C. Muldoon O. T. (2010). Socio-political context and accounts of national identity in adolescence. *Br. J. Soc. Psychol.* 49 583–599. 10.1348/014466609X475972 19891823

[B92] Sullivan P. (2012). *Data Preparation and Analysis. In Qualitative Data Analysis Using a Dialogical Approach.* London: Sage Publications, 64–80. 10.4135/9781446268391.n4

[B93] Tajfel H. (1981). *Human Groups and Social Categories: Studies in Social Psychology.* Cambridge: Cambridge University Press.

[B94] Tajfel H. Turner J. C. (1979). “An integrative theory of intergroup conflict,” in *The Social Psychology of Intergroup Relations*, eds Worschel S. Austin W. G. (Monterey, CA: Brooks/Cole).

[B95] Tizard B. Phoenix A. (2002). *Black, White or Mixed Race?* London: Routledge.

[B96] Townsend S. S. M. Markus H. R. Bergsieker H. B. (2009). My choice, your categories: the denial of multiracial identities. *J. Soc. Issues* 65 185–204. 10.1111/j.1540-4560.2008.01594.x

[B97] Utsey S. O. Abrams J. A. Opare-Henaku A. Bolden M. A. Williams O. III (2015). Assessing the psychological consequences of internalized colonialism on the psychological well-being of young adults in Ghana. *J. Black Psychol.* 41 195–220. 10.1177/0095798414537935

[B98] Verkuyten M. (1997). Discourses of ethnic minority identity. *Br. J. Soc. Psychol.* 36 565–586. 10.1111/j.2044-8309.1997.tb01150.x

[B99] Verkuyten M. (2001). ‘Abnormalization’ of ethnic minorities in conversation. *Br. J. Soc. Psychol.* 40 257–278. 10.1348/014466601164849 11446230

[B100] Verkuyten M. (2013). Justifying discrimination against Muslim immigrants: outgroup ideology and the five-step social identity model. *Br. J. Soc. Psychol.* 52 345–360. 10.1111/j.2044-8309.2011.02081.x 22074206

[B101] Vignoles V. L. Regalia C. Manzi C. Golledge J. Scabini E. (2006). Beyond self-esteem: influence of multiple motives on identity construction. *J. Personal. Soc. Psychol.* 90 308–333. 10.1037/0022-3514.90.2.308 16536653

[B102] Wamford-Lock C. G. (1907). *Mining in Malaya for Gold and Tin.* London: Carwither and Goodman.

